# Supplementary material for: Vaccination by Two DerG LEAPS Conjugates Incorporating Distinct Proteoglycan (PG, Aggrecan) Epitopes Provides Therapy by Different Immune Mechanisms in a Mouse Model of Rheumatoid Arthritis
Source: Vaccines (Basel). 2021 May 2;9(5):448. doi: 10.3390/vaccines9050448 (PMC8147650; doi:10.3390/vaccines9050448)
Supplement: Supplementary file 1 [file vaccines-09-00448-s001.zip › vaccines-1164795-supplementary.pdf]

Table S1. P values of arthritis scores of control and LEAPS vaccinated GIA mice from the introductory study (study 1)

		Treatment Groups					
Days After Vaccination		**PBS in ADJUVANT vs CEL-4000**	**PBS in ADJUVANT vs DerG-PG275cit**	**PBS in ADJUVANT vs CEL-4000 + DerG-PG275cit**	**CEL-4000 vs DerG-PG275cit**	**CEL-4000 vs CEL-4000 + DerG-PG275cit**	**DerG-PG275cit vs CEL-4000 + DerG-PG275cit**
	9	0.1052	*0.0168	*0.0244	0.3235	0.3974	0.8878
	11	*0.0292	*0.0292	*0.0412	0.3235	0.3974	0.8878
	14	*0.0114	**0.0016	**0.0032	*0.0139	*0.0244	0.8324
	16	****<0.0001	**0.0025	****<0.0001	0.3235	*0.0139	0.1389
	18	****<0.0001	***0.0006	****<0.0001	0.6214	*0.0139	*0.0487
	21	****<0.0001	***0.0002	****<0.0001	0.5725	0.1805	0.0573
	23	****<0.0001	0.0573	****<0.0001	0.1052	0.4378	*0.0168
	25	**0.005	*0.0244	***0.0005	0.4806	0.5725	0.2045
	28	***0.0001	**0.004	****<0.0001	0.3235	0.5725	0.1211
	30	***0.0003	**0.0062	****<0.0001	0.3593	0.3974	0.0783
	32	**0.002	*0.0244	***0.0002	0.3974	0.5255	0.1389
	35	*0.0114	0.0671	**0.0016	0.4806	0.5255	0.1805

The groups of GIA mice and administration of control and LEAPS vaccines are as described in the legends of Figures 1 and 3. The days following the administration of the first vaccine are indicated in the left column. The 2-way repeated measures ANOVA, followed by Fisher's LDS multiple comparison tests were used to compare each group with every other group. (n=8 mice per group, *p =< 0.05, **p = < 0.01, ***p = <0.001, ****p = < 0.0001).

Additional information. Visual inspection of the mice was done 3 times a week and arthritis scoring was performed as described in the Methods. Each time, 2 investigators scored the animals independently from each other and from investigators preparing and administering the formulated vaccines. Data was analyzed by another investigator (reviewer) who determined if two scores given by the evaluators were in agreement. If there was a difference in arthritis scores greater than 1 per mouse between the two sets of results, the evaluators were sent back for another scoring before recording could proceed. When in agreement, the arthritis score results were entered into spreadsheets and statistically analyzed by the reviewer.

Table S2. P values of arthritis scores of control and LEAPS vaccinated GIA mice from the follow-up study (study 2)

		Treatment Groups					
Days After Vaccination		PBS in ADJUVANT vs CEL-4000	PBS in ADJUVANT vs DerG-PG275cit	PBS in ADJUVANT vs CEL-4000 + DerG-PG275Cit	CEL-4000 vs DerG-PG275Cit	CEL-4000 vs CEL-4000 + DerG-PG275Cit	DerG-PG275cit vs CEL-4000 + DerG-PG275Cit
	7	**0.0099	0.1231	0.1861	0.2949	0.205	0.8253
	10	***0.0005	>0.9999	**0.0012	***0.0005	0.7827	**0.0012
	12	****<0.0001	0.1521	*0.0243	**0.0044	*0.0419	0.4081
	14	***0.0001	0.956	0.4081	***0.0001	**0.0026	0.3776
	17	****<0.0001	***0.0001	****<0.0001	0.0542	0.8685	*0.0367
	19	****<0.0001	****<0.0001	****<0.0001	0.1861	0.3776	*0.0279
	21	***0.0002	0.0614	****<0.0001	0.0614	0.4401	**0.0084
	24	***0.0002	**0.0099	****<0.0001	0.2254	0.0614	**0.0022
	26	**0.0012	*0.0116	****<0.0001	0.4734	0.2949	0.0782
	28	**0.0018	0.0986	**0.0044	0.137	0.7827	0.2254
	31	***0.0004	**0.0279	**0.0026	0.1684	0.5812	0.4081
	33	***0.0003	0.3486	**0.0037	**0.0072	0.4734	*0.0477
	35	***0.0004	0.2254	**0.0099	*0.0182	0.321	0.1684

The groups of GIA mice and administration of control and LEAPS vaccines are as described in the legends of Figures 1 and 3. The days following the administration of the first vaccine are indicated in the left column. The 2-way repeated measures ANOVA, followed by Fisher's LDS multiple comparison tests were used to compare each group with every other group. (n=8 mice per group, *p =< 0.05, **p = < 0.01, ***p = <0.001, ****p = < 0.0001).

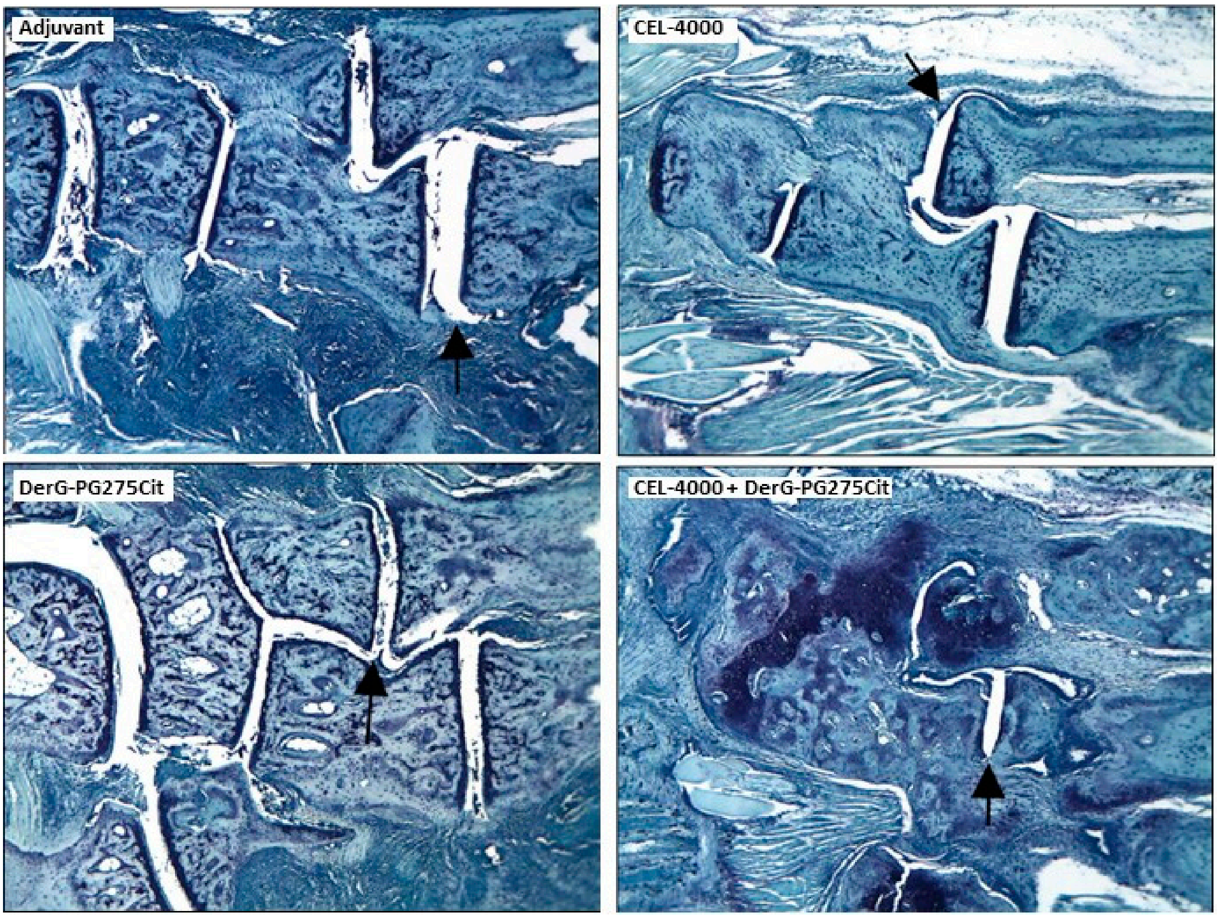

Figure S1. Representative histopathology images of ankle and foot joints of mice from the follow-up study (study 2). Sections of ankles and midfoot joints from adjuvant-treated control or LEAPS vaccine treated GIA mice (n=8 mice per group) were stained with toluidine blue. Each section was selected to reflect the average of the summed pathological score of each treatment group. The black arrow in each section point to one of the lesions found in the joints. The adjuvant-treated animal displays marked inflammation and cartilage damage, moderate synovial pannus formation and bone resorption as well as moderate periosteal bone formation. The CEL-4000-treated animal shows moderate inflammation and mild cartilage damage. The joints of the animal treated with DerG-PG275Cit show marked inflammation, moderate cartilage damage, minimal pannus and bone resorption as well as moderate periosteal bone formation. The animal treated with the combination of CEL-4000 and DerG-PG275Cit displays marked inflammation, moderate cartilage damage, mild pannus and bone resorption, and marked periosteal bone formation. Magnification: 40x. Inflammatory and structural damages, shown in the joint sections of the 4 treatment groups, were evaluated in the order of severity as follows: Adjuvant treatment (most severe), CEl-4000 + DerG-PG275Cit treatment (severe), DerG-PG275Cit treatment (moderately severe), CEL-4000 treatment (least severe). Detailed histopathology results are shown in Figure 4.

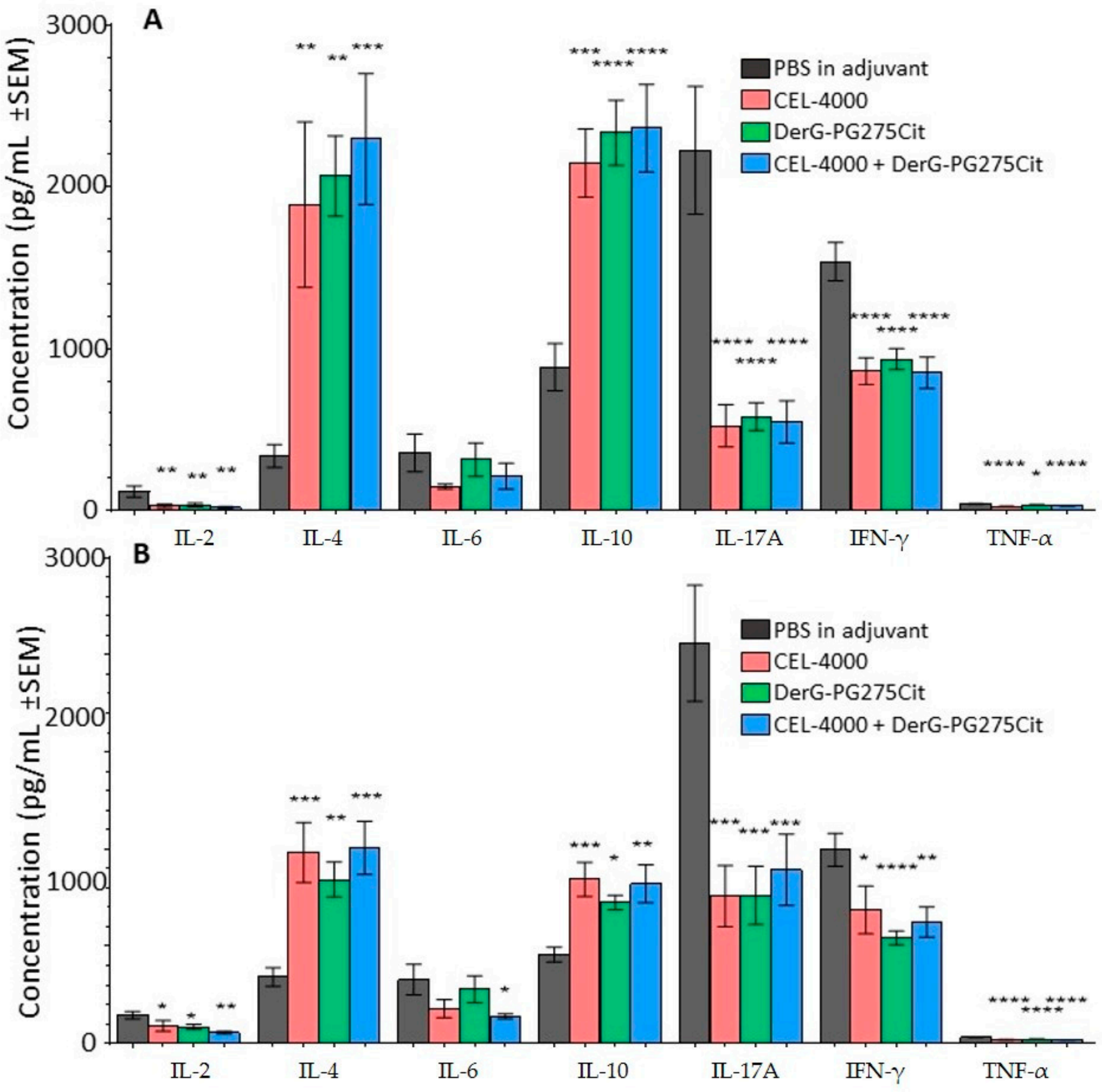

Figure S2. Concentrations of secreted cytokines in spleen cell culture supernatants from LEAPS vaccinated GIA mice. Cells were cultured **panel A)** in the absence of rhG1 antigen (in vitro non-stimulated cultures, or **panel B),** in the presence of rhG1 antigen (in vitro PG-stimulated cultures). Black bars, PBS + adjuvant-treated (Control); red bars, DerG-PG70 (CEL-4000) + adjuvant -treated; green bars, DerG-PG275Cit +adjuvant -treated groups; blue bars, DerG-PG70 (CEL-4000) + DerG-PG275Cit + adjuvant -treated groups. Culture conditions are described in detail in the Methods section. Results are expressed as the means ± SEM of cytokine levels (pg/ml) (n=8 mice per group) measured using a multiplex (Luminex MagPix) kit as described in the Methods. Data were analyzed using one-way ANOVA followed by comparisons between the control and each of the LEAPS-treated groups employing Fisher's LSD test. (*p =< 0.05, **p = < 0.01, ***p = <0.001, ****p = < 0.0001). Murine Cytokines evaluated were IL-2, IL-4, IL-6, IL-10, IL-17A, IFN-γ, TNF-α. Note IL-1β was below the limit of detection for the assay. For further details of cell isolation and stimulation see Materials and Methods.

Th Cell Subset-Peptide	Concentrations of Key Cytokines				Ratios of Anti- to Pro-inflammatory Cytokines			
	IL-4	IL-10	IFN-γ	IL-17A	IL-4:IFN-γ	IL-4:IL-17A	IL-10:IFN-γ	IL-10:IL17A
	pg/mL (Mean ± SEM)	pg/mL (Mean ± SEM)	pg/mL (Mean ± SEM)	pg/mL (Mean ± SEM)	Mean	Mean	Mean	Mean
Th0 - None	1032 ± 429	2550 ± 885	187 ± 45	38 ± 15	5.51	27.09	13.62	66.96
Th0 – CEL-4000	769 ± 291	2100 ± 885	429 ± 234	46 ± 8	1.79	16.89	4.9	46.14
Th1 - None	135 ± 44	365 ± 97	1504 ± 351	25 ± 5	0.09	5.32	0.24	14.4
Th1 – CEL-4000	121 ± 39	497 ± 134	1885 ± 225	29 ± 8	0.06	4.13	0.26	16.91
Th2 - None	926 ± 380	1797 ± 380	53 ± 28	63 ± 12	17.45	14.6	33.84	28.33
Th2 – CEL-4000	11865 ± 6039	9286 ± 1376	151 ± 74	60 ± 11	78.45	196.65	61.39	153.91
Th17 - None	107 ± 36	410 ± 132	69 ± 34	9145 ± 918	1.55	0.01	5.96	0.04
Th17 – CEL-4000	83 ± 26	479 ± 164	197 ± 71	12482 ± 1863	0.42	0.01	2.43	0.04

Figure S3. The effects of CEL-4000 on the *in vitro* differentiation of Th cell subsets from unvaccinated GIA mice. CD4+ spleen cells isolated from unvaccinated GIA mice were co-cultured with GIA spleen antigen-presenting cells, rhG1, and without (None) or with the CEL-4000 peptide (CEL-4000) in the presence of Th1, Th2, or Th17 differentiation promoting reagents. Following the removal of reagents on day 5 or 6, CEL-4000 and rhG1 were added back for a 2-day post-differentiation period and then the culture media were harvested for cytokine assays. Results are expressed as concentrations (pg/ml) of key cytokines (IL-4, IL-10, IFN-γ, IL-17A) (left side) or ratios of anti-inflammatory to pro-inflammatory cytokines (right side) detected in the culture media. The heat map at the right side illustrates the ratios of IL-4 and IL-10 to IFN-γ (red columns) or the ratios of IL-4 and IL-10 to IL-17A (green columns). Within each column, low cytokine ratios are represented by white (or light red or light green shades) and high ratios are depicted in dark red or dark green colors. Samples from 3 biological replicates were assayed, and data are expressed as means ± SEM or means.

Th Cell Subset - Peptide	Concentrations of Key Cytokines					Ratios of Anti- to Pro-inflammatory Cytokines			
	IL-4	IL-10	IFN-γ	IL-17A		IL-4:IFN-γ	IL-4:IL-17A	IL-10:IFN-γ	IL-10:IL17A
	pg/mL (Mean ± SEM)	pg/mL (Mean ± SEM)	pg/mL (Mean ± SEM)	pg/mL (Mean ± SEM)		Mean	Mean	Mean	Mean
Th0 - None	73049± 16002	13345 ± 1648	3014 ± 521	73 ± 33		24.24	1000.4	4.43	182.76
Th0 – CEL-4000	67923 ± 8422	13294 ± 1983	2888 ± 319	75 ± 29		17.49	676.51	4.6	178.1
Th1 - None	13837 ± 1616	2180 ± 483	25832 ± 6711	66 ± 34		0.54	210.05	0.08	33.09
Th1 – CEL-4000	12547 ± 1114	2529 ± 508	25912 ± 6672	68 ± 36		0.48	184.29	0.1	37.15
Th2 - None	145624 ± 19353	31986 ± 3695	2935 ± 597	76 ± 34		49.62	1,922.73	10.9	422.33
Th2 – CEL-4000	180698 ± 20398	30490 ± 4519	2632 ± 469	97 ± 41		68.66	1854.84	11.59	312.97
Th17 - None	338 ± 47	2049 ± 171	3256 ± 421	11351 ± 1995		0.1	0.03	0.63	0.18
Th17 – CEL-4000	352 ± 73	1879 ± 50	3318 ± 484	13135 ± 2839		0.11	0.03	0.57	0.14

Figure S4. The effects of CEL-4000 on the *in vitro* differentiation of polyclonally activated Th cell subsets from naïve unvaccinated mice. CD4+ spleen cells isolated from naïve unvaccinated mice were co-cultured with naive spleen antigen-presenting cells, activated with anti-CD3 and anti-CD28 antibodies, and cultured without (None) or with the CEL-4000 peptide (CEL-4000) in the presence of Th1, Th2, or Th17 differentiation promoting reagents. Following the removal of reagents on day 5 or 6, CEL-4000 and anti-CD3/CD28 antibodies were added back for a 2-day post-differentiation period and then the culture media were collected for cytokine assays. Results are expressed as concentrations (pg/ml) of key cytokines (IL-4, IL-10, IFN-γ, IL17A) (left side) or ratios of anti-inflammatory to pro-inflammatory cytokines (right side) detected in the culture media. The heat map at the right side illustrates the ratios of IL-4 and IL-10 to IFN-γ (red columns) or the ratios of IL-4 and IL-10 to IL-17A (green columns). Within each column, low cytokine ratios are represented by white (or light red or light green shades) and high ratios are depicted in dark red or dark green colors. Samples from 4 biological replicates were assayed, and data are expressed as means ± SEM or means.
